# Feasibility and Effect of the Exergame BOOSTH Introduced to Improve Physical Activity and Health in Children: Protocol for a Randomized Controlled Trial

**DOI:** 10.2196/24035

**Published:** 2020-12-11

**Authors:** Gabrielle ten Velde, Guy Plasqui, Maartje Willeboordse, Bjorn Winkens, Anita Vreugdenhil

**Affiliations:** 1 Department of Nutrition and Movement Sciences Maastricht University Medical Centre Maastricht Netherlands; 2 Maastricht University Maastricht Netherlands

**Keywords:** exercise, sedentary lifestyle, mHealth, mobile health, serious game, exergame, prevention, pupil, randomized controlled trial

## Abstract

**Background:**

Despite the well-known beneficial health effects of physical activity (PA), the majority of Dutch primary school children do not meet the recommended PA guidelines. Although there is growing evidence on the effectiveness of exergames for PA in children, there is limited evidence on their effect on health outcomes, such as cardiovascular health and health-related quality of life (HRQOL), and on factors influencing their effectiveness and feasibility. The exergame BOOSTH uses a wrist-worn activity tracker to measure steps per day. As a reward for the performed PA, children can unlock levels in the online BOOSTH game. In addition, “BOOSTH battle” enables competition between groups.

**Objective:**

This protocol describes a cluster randomized controlled trial in 16 primary schools in the Netherlands investigating the effect of BOOSTH on moderate-to-vigorous PA (MVPA) using accelerometry. Secondary aims are to investigate the feasibility of BOOSTH (mixed methods: questionnaires and focus group interviews) and its effect on cardiovascular risk factors (anthropometrics, blood pressure, and retinal microvasculature) and HRQOL.

**Methods:**

Stratification variables and relevant variables related to outcomes (such as BMI [z-score], sex, age, and parenting style) and/or missingness will be taken into account. Measurements will be performed at baseline and after 3, 6, and 12 months.

**Results:**

The study has received funding from Province Limburg (SAS-2015-04956) and received ethical approval from the Medical Ethics Committee of Maastricht University Medical Centre (METC172043/NL64324.068.17). The results of the analyses are expected to be published in 2021.

**Conclusions:**

With this study, the ability of the exergame BOOSTH to increase PA and improve health in children of primary school age will be investigated. The insights into effectiveness and feasibility will result in scientific and societal recommendations, which could potentially contribute to widespread implementation of exergames for children.

**Trial Registration:**

ClinicalTrials.gov NCT03440580; https://clinicaltrials.gov/ct2/show/NCT03440580.

**International Registered Report Identifier (IRRID):**

DERR1-10.2196/24035

## Introduction

Physical activity (PA) is associated with numerous health benefits in children [1], including the prevention of cardiovascular disease and obesity, and a better health-related quality of life (HRQOL) [2-4]. Moreover, higher levels of cardiorespiratory fitness in childhood are associated with a better cardiovascular health profile later in life [5]. Unfortunately, according to Jago et al, the levels of moderate-to-vigorous PA (MVPA) decrease with increasing age starting from the age of 6 years [6]. In 2019, 44.1% of Dutch children (age 4-11 years) and 59.5% of adolescents (age 12-18 years) did not meet the public health guidelines of performing a minimum of 60 minutes of MVPA each day [7]. Therefore, it is of major importance to promote PA from a young age.

Several studies have evaluated the effects of interventions aiming to increase PA on health outcomes in children (age 3-18 years) [4]. Most interventions showed improvements in HRQOL [4], physical fitness, and vascular structure in response to increasing PA [8,9]. Although the results of the majority of previous studies showed that higher PA in children (between the ages of 6 and 15 years) was associated with improved retinal microvascular health, the study by Lundberg et al is the only one that investigated objectively measured PA [8-10]. This study included 307 Danish children (mean age 15.4 years [SD 0.7]) and found that higher PA was associated with narrower retinal venules. Although questionnaires are easy to use, self-report methods may lead to recall bias. Especially in young children (age <10 years), questions on PA might be difficult to interpret or answer, and they may not fully understand the concept of PA. Longitudinal data on the effects of objectively measured PA on vascular function, sedentary behavior, and HRQOL and in the school-based population are scarce.

Nowadays, the majority of children spend a considerable amount of their time playing digital games, and as a result, the amount of screen time is high [11]. Among primary school children in the Netherlands, in 2015, 87% played digital games, of which 33% played on a daily basis [12]. Moreover, 53% of all children spend 1 to 4 hours on each gaming session, which highly contributes to sedentary time. However, the interest for video games also provides new opportunities to increase PA via exergames. An exergame is a game designed for a primary purpose beyond that of pure entertainment and aims to increase the player’s PA [13,14]. Exergames (ie, Wii Fit, Wii Sports, and Dance Dance Revolution) are videogames that combine PA with video gaming technology, thereby increasing energy expenditure and controlled body movements [15,16]. Previous studies have shown that exergames are more attractive and enjoyable for children in comparison with regular PA [17], suggesting that exergames are a promising tool to promote exercise in this population. Moreover, by improving the intrinsic motivation to perform PA, exergames might support healthier lifestyle habits in the long term [18].

To increase impact, exergames have recently been introduced in the school setting. This approach is promising, as children spend a considerable amount of time at school and children of all social economic classes are reached. The majority of school-based exergames showed enhanced PA [19-23]. For example, Gao et al showed that exergaming and regular physical education had similar effects on children’s (mean age 8.27 years [SD 0.70]) MVPA, light PA, sedentary behavior, and energy expenditure [22]. Exergame interventions have even more potential if they are applicable both within the school setting and during leisure time, since parental involvement increases success rates [24].

BOOSTH is a newly developed exergame that incorporates these principles. In order to unlock new levels in the online game, a child needs to be physically active (ie, take steps), thereby promoting the intrinsic motivation to move. BOOSTH makes use of a wearable device (activity tracker around the wrist) and can therefore be used within and outside the school setting, as well as during indoor and outdoor play.

This paper describes the protocol of a longitudinal approach to determine the effect of BOOSTH on PA (MVPA and sedentary behavior), BMI, cardiovascular risk factors (ie, blood pressure, retinal microvasculature, and maximal aerobic performance), and psychological parameters (ie, HRQOL and motivation towards PA) in a school-based setting that includes 16 schools in the Netherlands (BOOSTH study; ClinicalTrials.gov NCT03440580). Previous studies evaluating the effect of exergames focused mainly on PA, whereas measures of energy expenditure or BMI were included in a limited number of studies [17,21]. Stratification variables and relevant variables related to outcomes (such as BMI [z-score], sex, age, and parenting style) and/or missingness will be taken into account. Furthermore, in order to explain and interpret the findings and for future development of the BOOSTH intervention, the feasibility of implementation in a school setting will be investigated.

## Methods

### Recruitment and Participants

The BOOSTH study is a cluster randomized controlled trial at 16 primary schools located in the province of Limburg (the Netherlands). Schools will be randomized to standard physical education curriculum plus BOOSTH (intervention schools) or standard physical education curriculum only (control schools). Stratified randomization will be applied by an independent researcher. Stratification variables are community (ie, schools need to be located within the same community) and school size (ie, number of children in a school). Control schools will only be visited for physical measurements. The inclusion criteria for the children are as follows: boys and girls in classes five to seven (age range 7-12 years) at the moment of inclusion and written informed consent from both parents and children older than 12 years. Children will be excluded if they are wheelchair dependent.

### Sample Size Requirements

The sample size calculation is based on the primary outcome as follows: to detect a difference in MVPA between the intervention and control groups after 12 months. Children are nested within schools, with eight intervention and eight control schools.

Children from grades five till seven (age range 7-12 years) will be included in this study. Based on the participation rates of other Dutch school-based PA interventions, it is expected that about 60 children per school will participate. Therefore, 480 children per condition (intervention/control) (8×60=480) will be included. It is assumed that the intraclass correlation coefficient is between 0.05 and 0.10 [25]. When an intraclass correlation coefficient of 0.10 is used, the design effect (accounting for clustering of 60 children within a school) is equal to 6.9 (1+[60−1]×0.10). Accounting for a dropout rate of 20%, the effective sample size (sample size after dropout divided by design effect) is equal to 55 per school.

With the given sample size and assuming a significance level (α) of .05 and a power of 80%, we can demonstrate a Cohen *d* of 0.54, which means we can detect a moderate effect size on MVPA [26]. Based on a within-group SD of 20.38 [27], a difference in mean absolute MVPA of 11 min/day between the intervention and control groups can be detected.

### BOOSTH Intervention

The BOOSTH intervention consists of an online arcade game that can be synchronized with a BOOSTH activity tracker using the BOOSTH mobile app. In addition, the app includes “BOOSTH battle” to enable competition between groups.

After baseline measurements, children at the intervention schools will receive the BOOSTH activity tracker for free. The activity tracker, accompanied with a user manual and an oral instruction on how to use BOOSTH, will be provided to all children and parents during a regular school day. To install the BOOSTH app, a mobile device with Bluetooth and internet access is needed. Parents are asked to download the app together with their child. The children will receive an instruction to wear the BOOSTH activity tracker around their wrist during waking hours, except during water activities (eg, showering and swimming). One week after BOOSTH delivery, the research team will visit participating schools. Children, their parents, and their teachers will have the opportunity to ask questions about possible issues they encounter during the first week. No further instructions, activities, or support are part of the intervention.

The BOOSTH intervention includes several behavioral change techniques with the intention to stimulate PA and positively change the attitude toward PA. Using the BOOSTH activity tracker, the child can track step count. The use of activity trackers could help children set achievable goals and monitor their progress toward the goals [28,29]. The step count registered by the activity tracker can be synchronized with the BOOSTH app, which, depending on the magnitude of PA levels, is rewarded by online playtime and gaming incentives such as unlocked levels and special features. In order to unlock the next level, 30 minutes of PA are required (ie, about 6000 steps). A level in the BOOSTH game takes around 5 minutes to play. According to international guidelines, a maximum of 2 hours of screen time is recommended for children between the ages of 4 and 11 years [30]. Therefore, the amount of performed PA, which is needed to unlock the next level in the game, is six times higher compared with the amount of playtime. Rewards can motivate children to engage in PA, especially when combined with PA monitoring [31-33]. The first four levels are freely accessible in order to increase the interest and enthusiasm of the child. In addition, there is an optional function of a “BOOSTH battle” that enables competition between groups. Teachers and children can create their own battles in the BOOSTH app as they like.

### Measurements

#### Data Collection

**Figure 1:**
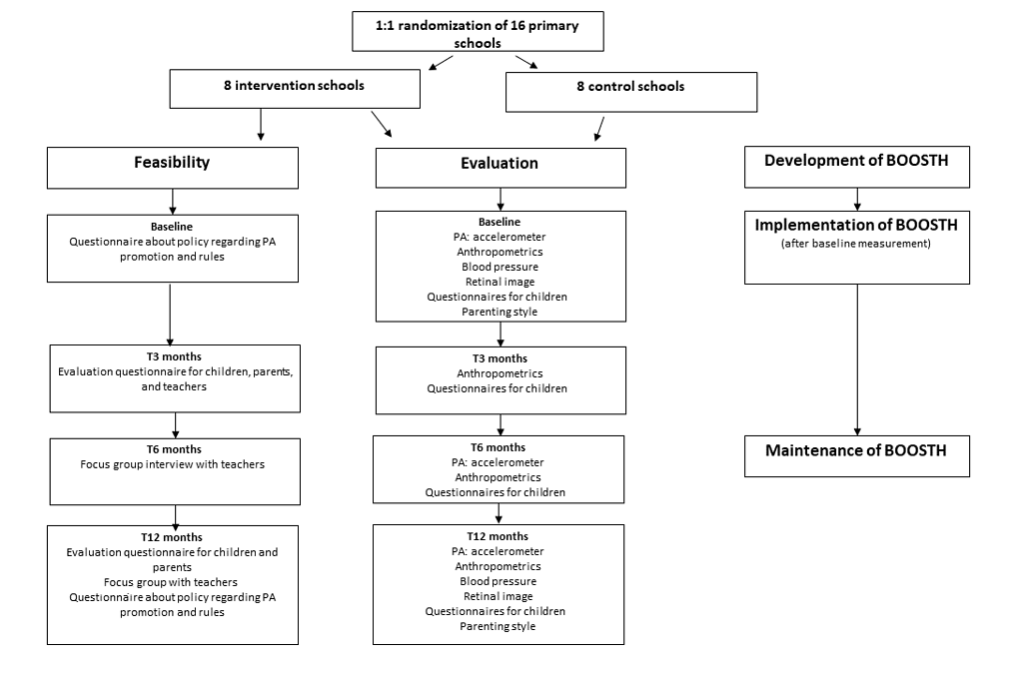
Overview of the BOOSTH study and measurements. The questionnaires for children included subjective PA behavior, screen time, health-related quality of life, and motivation toward PA. PA: physical activity.

As shown in Figure 1, study measurements will be performed at baseline (T0) and after 3 months (T3), 6 months (T6), and 12 months (T12). A trained research team will perform the measurements during school hours at the primary schools. The research team will be instructed to complete the questionnaires together with the children. Stratified intervention and control schools will be measured within the same week to prevent confounding effects of, for example, different seasons and weather-dependent factors influencing PA. Measurements will be performed using a standardized protocol.

#### Physical Activity

The primary outcome is MVPA (min/day), which will be measured with Actigraph GT3X (ProCare). This is a triaxial accelerometer, and it has been validated for the measurement of PA behavior in children. The children will be asked to wear the accelerometer attached via a waistband on the right hip for 7 consecutive days during waking hours, except during water activities (eg, showering and swimming) and contact sports (eg, judo). Accelerometry data will be downloaded with 10-s epochs using the Actilife software (Actigraph). Valid wear time will be defined as a minimum of 4 days, with at least 480 minutes per day of recording, including 1 weekend day. Derived data are expressed as mean counts per minute (cpm). To establish time spent in different intensity categories (sedentary behavior, light PA, and MVPA), the cutoff points developed by Everson et al will be used [34].

In addition, the self-administered validated Baecke questionnaire (for children) will be used to assess the amount of habitual PA ranging from 1 (lowest activity) to 5 (highest activity) [35]. Data on PA at school, during leisure time, and during organized sports will be collected.

#### Anthropometrics

Height, weight, and waist circumference will be measured in duplicate, and the average of both measurements will be reported. Body weight will be measured using an electric scale (Seca 877, Seca) to the nearest 0.1 kg. Heavy clothing and shoes will be removed before the measurement. Standing height will be measured using a portable stadiometer (Seca 213 stadiometer, Seca), and children will be asked to stand straight and look forward. Subsequently, BMI (weight [kg]/height [m]^2^) will be calculated and age- and sex-specific BMI z-scores will be calculated (TNO Growth Calculator, TNO) [36]. In addition, weight classifications will be performed using the International Obesity Task Force classification system [37]. Moreover, waist circumference will be measured using a nonelastic tapeline after normal exhalation. Height and waist circumference will be measured to the nearest 0.01 m.

#### Cardiovascular Risk Factors

Systolic and diastolic blood pressure will be measured using an automated sphygmomanometer (Mobil-O-Graph, Revision 4.6 08/2011, IEM GmbH). Blood pressure will be measured on the nondominant arm in the sitting position. Measurements will be performed thrice, with 1 minute of rest in between measurements. The reading will be recorded to the nearest 1 mmHg. A mean value will be calculated out of these three readings. The mean systolic and diastolic blood pressure values will be computed into z-scores using the LMS method (L, curve Box-Cox; M, curve median; S, curve coefficient of variation) and reference values as described by Wühl et al [38,39].

Retinal microvasculature will be measured with fundus photography, which is a noninvasive tool to measure cardiovascular risk factors. The retinal characteristics that have received the most attention so far in predicting cardiovascular disease are the central retinal arteriolar equivalent (CRAE) and the central retinal venular equivalent (CRVE). Photographs of the retinal microvasculature will be taken using a fundus camera (Topcon TRCNW-300, Topcon Corporation). Subjects will be seated with their head resting on a chinrest, thereby looking straight in the camera. The fundus camera automatically focuses on the subject’s pupils and will take a photo of the retina of the right eye. A fundus photo with a centralized optic disc will be obtained for each subject. Fundus photos will be analyzed using RetinaCheck, and manual modification will be done using the retinal health information and notification system (RHINO). These programs are developed in collaboration with the Department of Biomedical Engineering at Eindhoven University of Technology in the Netherlands, using a series of innovative brain-inspired algorithms as described by ter Haar Romeny et al [40]. The region between two times and three times the radius of the optic disc will be used to calculate the CRAE and CRVE, and to calculate the ratio between the CRAE and CRVE (arteriolar-to-venular diameter ratio).

The maximum aerobic performance of the children will be assessed with a maximum multistage 20-m shuttle run test. Children run back and forth on a 20-m course and have to touch the 20-m line before or at an audio signal that is emitted from a prerecorded tape. The frequency of the audio signal is increased by 0.5 km/h each minute from a starting speed of 8.5 km/h. The test ends when the child has to stop owing to fatigue or when the child fails to reach the 20-m line concurrent with the audio signal on two consecutive occasions. The children will be constantly encouraged to run for as long as possible throughout the course of the test. The last completed oxygen uptake (VO_2max_) (Y, mL/kg/min) will be calculated from the speed (X, km/h) corresponding to that stage (speed=8+0.5 stage number) and age (A, year) as follows: Y=31.025+3.238X−3.248A+0.1536AX [41].

#### Screen Time

Self-reported screen time will be reported separately for weekdays and weekend days using the following questions: “How many hours a day during the last 4 weeks did you watch TV on a normal weekday/weekend day?” and “How many hours a day during the last 4 weeks did you play console games or use a computer for your free time activities on a normal weekday/weekend day?” Possible responses are “not at all,” “0.5 hours per day,” “one hour per day,” “2 hours per day,” “2.5 hours per day,” “3 hours per day,” “3.5 hours per day,” and “4 hours or more per day.” Total screen time will be reported as minutes spent on watching television plus computer use. At 3, 6, and 12 months, a question about changes in screen time since the introduction of BOOSTH will be included in the questionnaire.

#### Psychological Parameters

HRQOL will be assessed using the validated Kidscreen and validated Pediatric Quality of Life Inventory (PedsQL) questionnaires. The 23-item PedsQL addresses the multidimensional scales of physical, emotional, social, and school functioning. The questionnaire consists of five options ranging from “never” to “almost always” [42]. Additionally, the 27-items Kidscreen will be used to measure HRQOL for five dimensions (physical well-being, psychological well-being, autonomy & parent relation, peers & social support, and school environment) [43].

Motivation toward PA will be measured using the self-administered validated Behavioral Regulation in Exercise Questionnaire-2 (BREQ2). The BREQ2 consists of 19 questions with a 5-point scale, which measures different aspects of motivation (external regulation, identification, introjection, integration, motivation, and intrinsic regulation) and has been shown to have good factorial validity [44].

#### Parenting Questionnaires

Parents will be asked to fill in an online questionnaire regarding family composition, country of birth, educational level, and self-reported height/weight. In addition, parents will be asked to fill in the validated 85-item comprehensive general parenting questionnaire (CGPQ). The CGPQ assesses the following five key parenting constructs that have been identified across multiple theoretical approaches of parenting: “nurturance,” “structure,” “behavioral control,” “coercive control,” and “overprotection” on a five-factor scale [45].

The parent version of the validated Kidsscreen-27 questionnaire will be used to determine a HRQOL score of the child, and it consists of 27 questions. These questions are similar to the Kidsscreen-27 child version [43].

#### Feasibility

To understand which components of the intervention are successful, feasibility of the BOOSTH intervention will be assessed using a mixed-methods design including semistructured focus groups with teachers and evaluation questionnaires. In addition, the strengths, limitations, opportunities, and recommendations for future development will be asked.

Semistructured focus group interviews will be performed with teachers from the intervention schools. Prefixed interview guides will be used, with the possibility of additional follow-up questions, which allows the researcher to cover both contextual factors and intervention implementation. Questions will be asked about the introduction lesson of BOOSTH, the implementation and stimulation of BOOSTH by the teachers, and suggestions for future development of BOOSTH.

Children and their parents will be asked to fill in an evaluation questionnaire about their experiences with BOOSTH. Teachers will be asked to fill in an evaluation questionnaire about the implementation of the BOOSTH intervention.

#### BOOSTH Step Count

Children who are allocated to the intervention schools will receive the BOOSTH activity tracker. The BOOSTH activity monitor is a lightweight triaxial accelerometer–based activity monitor, which is worn around the wrist and measures the step count. Moreover, the BOOSTH activity monitor resets automatically at midnight and stores data of the previous day automatically on the device. Data are stored on the device for 30 days. A child or parent has to synchronize the BOOSTH activity monitor with a smartphone or tablet to retrieve the measured step count. The BOOSTH activity tracker will be used as a motivation tool to promote PA. The step count on the BOOSTH activity tracker will not be used for research purposes.

### Statistical Analysis Plan

Numerical variables will be presented as mean and SD or median and IQR where appropriate. Categorical data will be presented as number and percentage of participants in each of the possible categories. Baseline characteristics will be analyzed for differences between the groups, using independent sample *t* tests or Mann-Whitney *U* tests for numerical variables and chi-square or Fisher exact tests for categorical variables. The effects of BOOSTH on numerical outcome parameters (such as MVPA, cardiovascular parameter, and HRQOL) will be analyzed using linear mixed models in order to correct for dependent observations owing to repeated measurements within a child and clustering of children within a school. Stratification variables and relevant variables related to the outcomes (such as BMI [z-score], sex, age, and parenting style [measured with the CGPQ]) and/or missingness will be taken into account. A random intercept on school level and an unstructured covariance structure for repeated measures will be included. A *P* value <.05 will be considered statistically significant. All analyses will be performed using IBM SPSS Statistics for Windows version 25.0 (IBM Corp).

The feasibility of the study will be investigated using a mixed-methods design. Qualitative data of the focus group interviews will be audio recorded and transcribed verbatim. Interview transcripts will be coded by themes and concepts using NVivo version 12 software (QSR International). Coding will be performed by two researchers.

## Results

The study has received funding from Province Limburg (SAS-2015-04956) and received ethical approval from the Medical Ethics Committee of Maastricht University Medical Centre (METC172043/NL64324.068.17). In September 2018, the inclusion procedure was started, and 16 primary schools were included and randomized into intervention school (n=8) and control school (n=8) groups. The results of the analyses are expected to be published in 2021.

## Discussion

The observation of children spending a considerable amount of their time on gaming creates a window of opportunity to use gaming to increase their PA. To promote PA throughout the day, the combination of an activity tracker and an exergame is a promising and innovative intervention. It seems that exergames are more attractive and enjoyable for children in comparison to regular PA, which could be the result of improving the intrinsic motivation of children to perform PA [17,18]. Studies evaluating the effects of exergames on PA and influencing factors are important to obtain more insights into the possibility of using these tools for health promotion. More importantly, information on the effects of exergaming on cardiovascular risk factors and other health parameters is scarce. The majority of previous studies that investigated the effect of PA on cardiovascular risk factors or other health parameters like HRQOL used self-reported measures for PA behavior. There is a need to confirm these results with objectively measured PA behavior, especially for cardiovascular risk factors such as retinal microvasculature [8,9]. BOOSTH is an exergame that combines an activity tracker, which measures step count, and an online jump-and-run game as a reward for the performed PA. BOOSTH takes a novel approach since it could be incorporated both inside and outside the school setting to have even more potential. Moreover, in contrast to other exergames that combine the PA element with the game (eg, Nintendo Wii), BOOSTH is an exergame in which users need to perform PA in the real world before they can play the BOOSTH game. The aim of this study is to evaluate whether an intervention with BOOSTH promotes PA, health, and HRQOL in primary school children. In addition, the feasibility of the BOOSTH intervention in a school setting will be investigated. The insights gained from the results of this study will enable us to formulate recommendations for future strategies for increasing PA in children at this crucial age. In addition, if the results of this study show that the exergame BOOSTH has a positive effect on PA levels and health of children, the implementation could be widespread as a unique and innovative addition to current PA activity interventions.

## Abbreviations

BREQ2: Behavioral Regulation in Exercise Questionnaire-2
CGPQ: comprehensive general parenting questionnaire
CRAE: central retinal arteriolar equivalent
CRVE: central retinal venular equivalent
HRQOL: health-related quality of life
MVPA: moderate-to-vigorous physical activity
PA: physical activity
PedsQL: Pediatric Quality of Life Inventory
